# Kinetics of Clobetasol-17-Propionate in Psoriatic Lesional and Non-Lesional Skin Assessed by Dermal Open Flow Microperfusion with Time and Space Resolution

**DOI:** 10.1007/s11095-016-1960-y

**Published:** 2016-06-06

**Authors:** Manfred Bodenlenz, Christian Dragatin, Lisa Liebenberger, Bernd Tschapeller, Beate Boulgaropoulos, Thomas Augustin, Reingard Raml, Christina Gatschelhofer, Nathalie Wagner, Khaled Benkali, Francois Rony, Thomas Pieber, Frank Sinner

**Affiliations:** 1 HEALTH - Institute for Biomedicine and Health Sciences, Joanneum Research Forschungsgesellschaft m.b.H, Neue Stiftingtalstrasse 2, 8010 Graz, Austria; 2 Division of Endocrinology and Diabetology, Department of Internal Medicine, Medical University of Graz, Auenbruggerplatz 15, 8036 Graz, Austria; 3 Galderma R&D, Sophia Antipolis, France

**Keywords:** dermal pharmacokinetics, lipophilic drug, open flow microperfusion, skin penetration, topical formulation

## Abstract

**Purpose:**

To evaluate the kinetics of topically applied clobetasol-17-propionate (CP-17) in lesional and non-lesional psoriatic skin when released from a commercially available low-strength cream using *in vivo* dermal open-flow microperfusion (dOFM).

**Methods:**

Twelve patients received Dermovate® cream (CP-17, 0.05%) on small lesional and non-lesional skin test sites for 14 days, once daily. On day 1 and 14, dOFM samples were continuously taken in the dermis for 24 h post-dose and analyzed by LC-MS/MS. Probe depths were assessed by 50 MHz ultrasound scanning.

**Results:**

Mixed-effects modelling identified skin condition, treatment duration and probe-depth as kinetics determining variables. The time- and depth-resolved intradermal data revealed (i) slower penetration of CP-17 into lesional than into non-lesional skin, (ii) normalized (faster) skin penetration after repeated dosing, and (iii) no CP-17 accumulation within the dermis independently of the skin condition.

**Conclusions:**

Intradermal investigation of a highly lipophilic drug released from low-strength cream was successfully performed by using dOFM and timely and spatially, i.e., probe-depth dependent, resolved kinetic data were delivered. These data support the assumption that the thickened psoriatic stratum corneum might act as trap compartment which lowers the skin penetration rate for lipophilic topical drugs.

**Electronic supplementary material:**

The online version of this article (doi:10.1007/s11095-016-1960-y) contains supplementary material, which is available to authorized users.

## Introduction

Topical corticosteroids play a major role in the therapy of inflammatory skin disorders (1, 2). These topically applied steroids are highly effective, but their delivery into the skin is sometimes rather inefficient (2). Additionally, the role of the stratum corneum (SC) in different skin diseases regarding the kinetics of such topically applied lipophilic drugs remains unclear up to now, because *in vivo* sampling of such drugs is still challenging. Sufficiently sensitive, accurate and well-tolerable *in vivo* sampling methods are not commonly available (3). Several existing *in vivo* sampling methods, such as punch biopsies (4), suction blister (4), or tape stripping (5, 6) are either highly invasive or do not provide any information on the drug concentration from the site of action. Dermal microdialysis (MD) (7) is minimally invasive and capable of directly measuring the rate and extent of drug absorption at or near the site of action in the skin (8), but for intradermal sampling of topically applied drugs, MD has certain limitations. Typical topically applied drugs are lipophilic and commercially available topical formulations contain potent active ingredients in low concentration, resulting in concentrations of the recovered drug in the MD sample that may even lie below the limit of quantification (9). Furthermore, the pore size of the semipermeable MD membrane restricts sampling of molecules by excluding large molecules. Open flow microperfusion (OFM) (10), in contrast to microdialysis, uses a fully permeable open mesh and has been adapted for dermal sampling (11, 12). It overcomes the limitations of MD when sampling lipophilic topical drugs (11, 13) and is thus capable of assessing topical glucocorticoid kinetics, when released from a commercial low-strength cream, *in vivo*.

Clobetasol-17-propionate (CP-17) was used as study drug. CP-17 is a highly potent corticosteroid for topical treatment of psoriasis (2, 14) and commercially available as cream with low strength (0.05%). Although CP-17 is widely used, data on the penetration behavior of this drug into skin is rare. One study performed by Au *et al*. (15) investigated the feasibility of CP-17 sampling in the skin, but they applied highly concentrated CP-17 in ethanolic solution, because the high lipophilicity of CP-17 (*LogP* = 3.49) precluded the use of a commercial formulation for MD sampling. A study on the penetration behavior of a topical lipophilic drug into healthy and psoriatic lesional skin using tape-stripping followed by skin punch biopsy was performed by Rony *et al*. (unpublished data, presented at Groupe de Métabolisme et Pharmacocinétique Meeting 2011, Paris). Their results revealed diminished drug penetration into psoriatic skin due to accumulation of the drug in the SC. Thus, they assumed that the thickened SC might act as trap compartment of the skin for lipophilic entities. Also in a previous dOFM study in psoriatic patients, using a moderately lipophilic drug, concentrations of the anti-psoriatic drug candidate BCT194 were found to be lower in psoriatic lesional skin than in non-lesional skin (12).

The aim of the current study was to evaluate the kinetics of highly lipophilic topical CP-17 *in vivo* in lesional and non-lesional psoriatic skin when released from a low strength cream and to elucidate the role of the SC in psoriasis by using timely and spatially, i.e., probe-depth resolved kinetic data.

## Materials and Methods

### Topical Treatment

Clobetasol-17-propionate (CP-17) is a highly potent corticosteroid (US class I, Europe class IV) that activates glucocorticoid receptors and exhibits anti-inflammatory and immunosuppressive activities. CP-17 is widely used for topical treatment of psoriasis (14) and it was applied as commercial cream of low-strength (Dermovate® Cream, 0.05% CP-17, GlaxoSmithKline Pharma GmbH, Vienna, Austria).

Dermovate® Cream was applied once daily from day 1 to day 14 to one lesional and one non-lesional test site of 7.7 cm^2^ each at a topical dose of 15 mg/cm^2^. On days 1 and 14, when dOFM sampling was continuously performed from baseline to 24 h post-dose, the cream remained on the skin and the test site was protected by a non-occlusive dressing. On the days without investigation by dOFM, the topical dose was applied once and excess of cream was removed at approximately 4 h post-dose.

### 24 h Dermal OFM Sampling

Continuous intradermal sampling was initiated and performed as described in (12, 16), by using CE-certified dOFM materials. The dOFM probe was tested for potential unspecific adsorption of CP-17 and the absence of any adsorptive losses was confirmed.

**Fig. 1:**
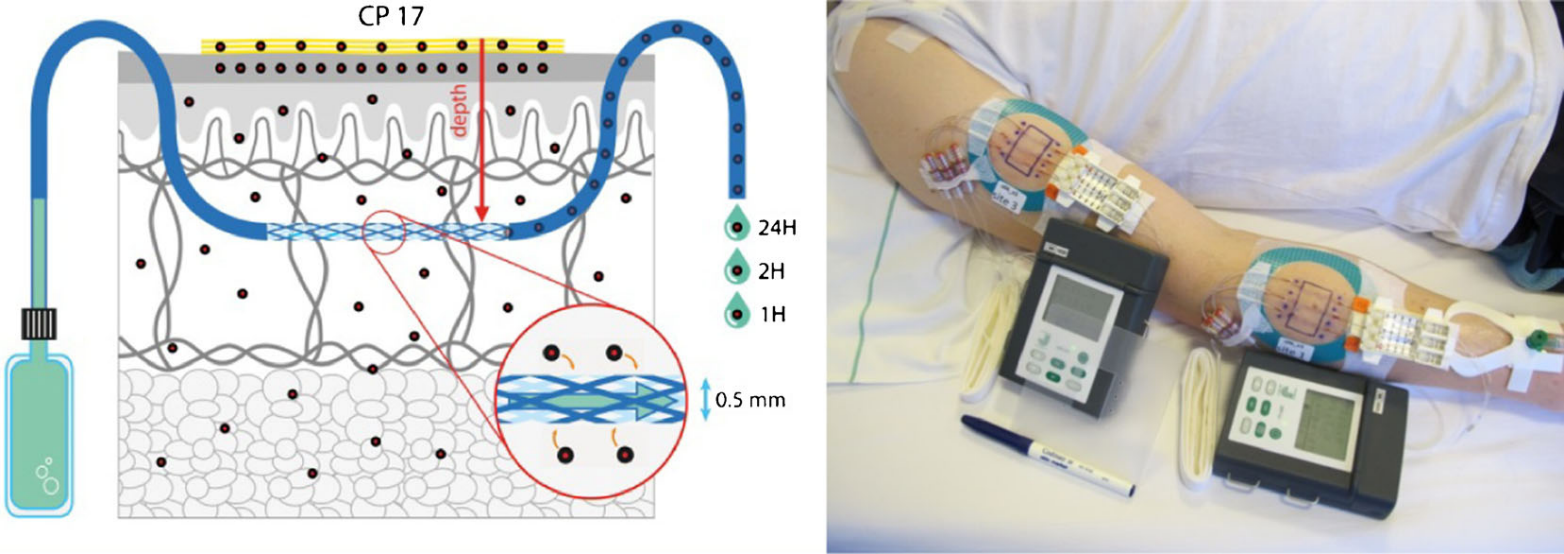
Left: Scheme of dOFM setup. The membrane-free dOFM probe within the dermis is continuously perfused and delivers interstitial fluid for further analysis. Right: Wearable dOFM setup including the dOFM probe, a wearable push-pull pump, and a unit for sample collection.

Prior to probe insertion, the skin was cooled with an sterile ice bag to avoid pain (16). Three linear dOFM sampling probes (Type DEA15001, OD 0.32 mm, 15 mm open-mesh with integrated 0.5 mm insertion needle) were inserted into a lesional test site and three probes into a non-lesional test site. Following insertion, the three adjacent probes were continuously perfused in a push-pull manner with sterile perfusate (physiological saline solution including 1% Human Serum Albumin) at a flow rate of 1 μL/min using a single wearable OFM pump (Type MPP101 with sterile fluidic kit). The dermal interstitial fluid samples were collected hourly from baseline to 24 h post-dose. The dermal OFM setup is shown in Fig. 1.

### Patients

The study was approved by the Ethical Committee of the Medical University of Graz and the Austrian health authorities (AGES) and performed in accordance with the Declaration of Helsinki and Good Clinical Practice. Twelve psoriatic patients with a diagnosis of stable plaque type psoriasis for at least 6 months and at least two psoriatic plaques on the upper extremities or proximal lower extremities were recruited (Caucasian, 9 men and 3 women, 40.2 ± 5.8 years) and gave written informed consent. The plaque Total Sum Score (TSS; erythema + induration/plaque elevation + scaling) had to be ≥ 6 with each item scoring 2 or 3 (moderate to severe) with a maximum of 2 for scaling. Subjects were otherwise healthy and screened for eligibility by assessing their medical history, physical parameters and laboratory parameters. Concomitant medication was not allowed, and women had to use contraception.

### Study Design

**Fig. 2:**
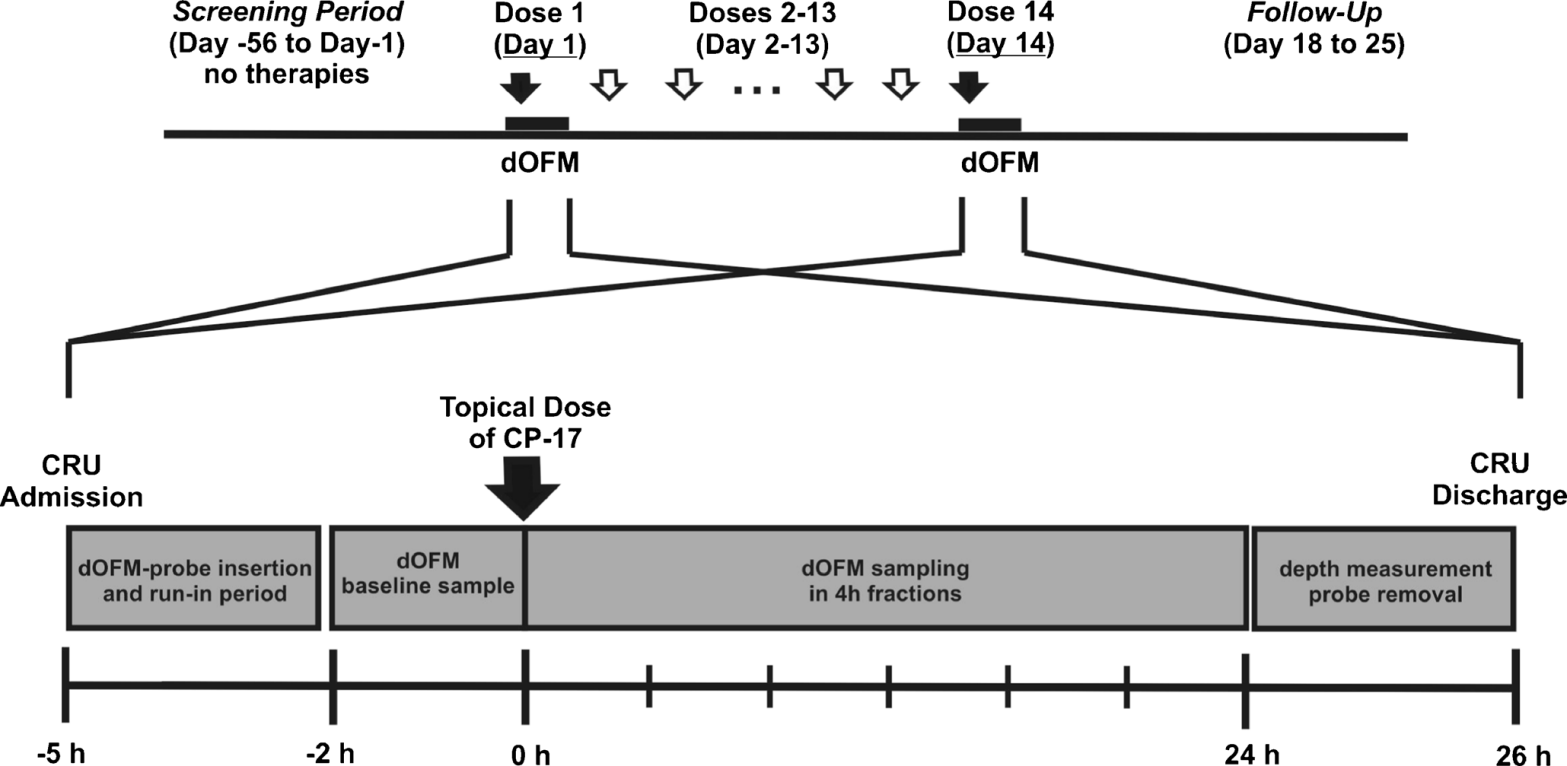
Study days with treatment regimen and protocol on the days of OFM investigation. CP-17 was dosed as Dermovate® Cream 0.05% once daily from day 1 to day 14. Following the doses on day 1 and day 14 the kinetics of CP-17 were followed in dermal interstitial fluid for 24 h by using dOFM.

The study was designed as a single-center, open-label exploratory phase I trial and conducted at the Medical University of Graz, Austria. In the pilot phase, topical CP-17 penetration was investigated in four subjects to identify the quantification limit for CP-17 from dOFM samples. In the main clinical study, eight subjects were dosed with CP-17 from day 1 to day 14, daily. dOFM was performed on day 1 and day 14. Patients were admitted to the clinic at 7 am (day 1). One lesional and one non-lesional site were marked on the skin, and three dOFM probes were inserted into the dermis of each site for continuous sampling as described above. After a run-in period of 60 min to allow the insertion trauma to subside (17), a baseline (pre-dose) sample was collected. At 11 am (*t* = 0), Dermovate® cream was applied to each site, as described above. dOFM samples were collected hourly, immediately frozen at −80°C and kept at −80°C until analysis. Sampling was terminated 24 h post-dose. Probe depth and the exact position in the skin were assessed in duplicate by 50 MHz ultrasound scanning (DUB-USB, Taberna Pro Medicum, Lüneburg, Germany) as described in detail previously (16). After probe removal, sites were inspected and covered with light dressings. On days 3–13, patients revisited the study center once daily, received the topical dose, left the clinic 1 h post-dose and were asked to remove the excess of cream approximately 4 h post-dose. On day 14, patients were admitted to the clinic in the morning. The same protocol as on day 1 was followed, but with two methodological alterations taking the reinsertion of dOFM to the now previously used test sites into account: First, the probes were shifted by 5 mm relative to their position on day 1. Second, the probe insertion points outside of the application zones were cleaned thoroughly with gauze, followed by slight tape stripping to reduce risk of probe contamination with residual drug in the SC. On day 14, the clinical improvement of CP-17 treated lesions was also evaluated using the plaque Total Sum Score (TSS). The follow-up visit was scheduled between days 18 and 25. An overview of the study days and procedures is given in Fig. 2.

### Sample Analysis and Data Management

Baseline sample and six pooled post-dose samples (0–4 h, 4–8 h, 8–12 h, 12–16 h, 16–20 h, 20–24 h) were analyzed to assess intradermal kinetics. CP-17 was measured after liquid-liquid extraction by LC-MS/MS in selected multiple-reaction monitoring (MRM) positive ion mode. Internal standard (CP-17-d3), 15 mM NH_4_OH (50 μL), and diethylether/hexane 80/20 (400 μL) were added to 50 μL of standards or samples. The samples were vortexed and centrifuged. Then, the organic layer was removed and evaporated to dryness under vacuum. The residue was redissolved in 30% acetonitrile (20 μL) and subsequently analyzed. The measurement was performed on a U300 Ultimate HPLC system (Dionex) coupled to a TSQ Ultra AM triple quadrupole mass spectrometer (Thermo). The separation was carried out on a Zorbax SB-C18 (0.5 × 35 mm) column (Agilent), with a flow rate of 50 μL/min and 7.5 μL injection volume using gradient elution (eluent A: water 0.1% formic acid; eluent B: acetontrile 0.1% formic acid). The gradient started with 40% eluent B which was linearly increased to 90% within 2.0 min and kept constant for until 4.5 min. The analyte was detected in MRM mode (CP-17: 467.2−446.9 (3 eV), 467.2−372.9 (9 eV), 467.2−354.9 (10 eV), 467.2−277.9 (17 eV), 467.2−262.9 (24 eV); Internal standard: 470.2−372.8 (8 eV), 470.2−354.8 (10 eV), 470.2−277.90 (18 eV), 470.2−262.8 (17 eV). The Lower Limit of Quantification (LLOQ) was 0.35 ng/mL.

For data management, we used an electronic case report form (eCRF) within the OpenClinica Enterprise Edition (OpenClinica, LLC 460 Totten Pond Road, Suite 200, Waltham, MA 02451). OpenClinica had been fully validated and it is 21 CFR Part 11 compliant.

### Pharmacokinetic Analysis and Statistics

Only the 8 subjects of the main study were included in the pharmacokinetic analysis. CP-17 concentration profiles were checked for validity, considering the concentration gradient from the skin surface (500 μg/ml) to the dermal interstitial fluid (about 0.5 ng/ml) and the risk of probe contamination with residual topical drug in the SC during reinsertion of probes on day 14. For the pharmacokinetic analysis, the areas under the CP-17 concentration time curves (AUCs) were calculated by cumulating sample concentrations from 0 to 24 h or, in case of a missing sample, by the standard trapezoidal method.

Outliers in CP-17 profiles were excluded when the outlying value was <50% or >200% of the interpolated concentration. All data were expressed as arithmetic mean ± SD. All AUC-values were log-transformed prior to data analysis. The log-transformed AUC-values were tested for statistical significance by means of two-factorial repeated measure ANOVAs using “Time” (“Day 1” *versus* “Day 15”) and “Skin site” (“Lesional” *versus* “Non-Lesional”) as within-subjects factors. P-values <0.05 were considered statistically significant. In case of a statistically significant ANOVA, paired t-tests were performed to find the significant mean differences. For the interpretation of these post-hoc tests on the raw data (i.e., non-depth corrected data) the significance level was adjusted according to Bonferroni. In order to keep the familywise error rate at 0.05, we adjusted the significance level to 0.05/4 = 0.0125. By applying this Bonferroni adjustment we followed a very conservative approach.

In order to analyze the effect of data treatment, the AUCs were calculated from three different data sets: Data set 1: All CP-17 raw data values below LLOQ were left unchanged; Data set 2: All CP-17 values below LLOQ were set to LLOQ; Data set 3: All CP-17 values below LLOQ were set to LLOQ/2.

The dependency of CP-17 concentration on the depth was identified in a stepwise approach: We (i) plotted the AUC data *versus* probe-depth, (ii) searched for adjacent probes with different depths and counted the pairs for which the AUC-depth relationship was true in a frequency analysis, and (iii) compared the probe with the greatest and the smallest depth per site with respect to the AUC, using paired t-tests. Finally, a linear mixed effects model (iv) was fitted to the data, using Log(AUC) as dependent variable, “Time” and “Skin site” as fixed effects, “Subject” as random effect, and “Probe-depth” as covariate. Model simplification was performed by using likelihood ratio tests, starting with the full model containing all predictor variables.

The open source software package “R” (Version 2.10.1) was used for the statistical analysis.

## Results

### CP-17 Treatment Effect and dOFM Tolerability

Study procedures and 24 h sampling periods were well tolerated by all subjects. No subjects dropped out and no serious adverse events occurred. Daily topical treatment with CP-17 resulted in a visible improvement of psoriatic skin within 14 days. The plaque Total Sum Score (TSS) for the lesional test site was reduced from 6.3 ± 0.5 on day 1–2.4 ± 1.4 on day 15 (*p* < 0.001) and appeared as a pale rectangular area within the otherwise unchanged psoriatic plaque.

### CP-17 Profiling by dOFM

**Fig. 3:**
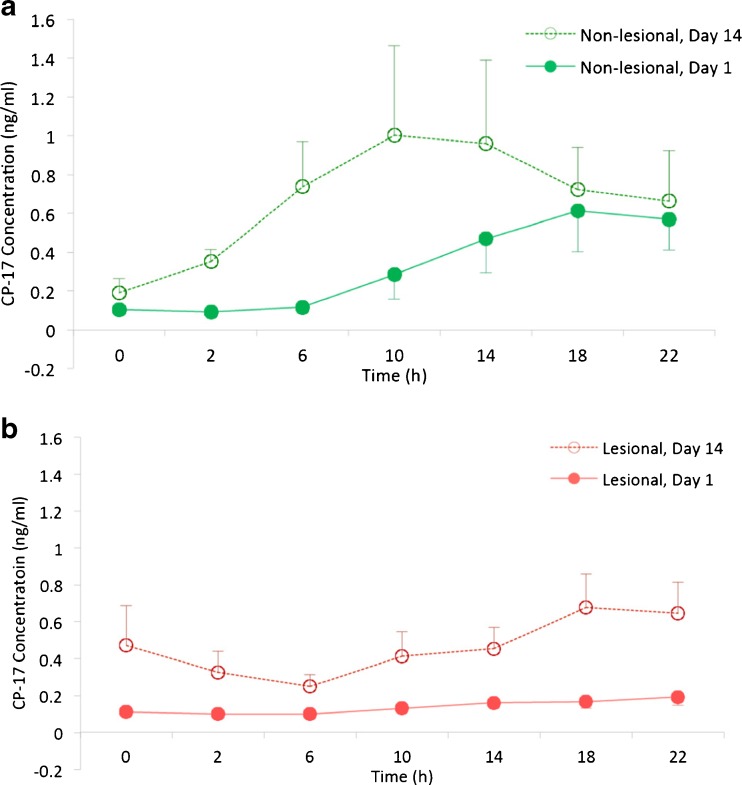
Mean CP-17 concentration profiles from baseline to 24 h post-dose on the Day 1 (after 1st dose) and Day 14 (after 14th dose). (**a**) Non-lesional skin profiles. (**b**) Lesional skin profiles. Data are mean ± sem.

On day 1, CP-17 concentrations in the dermal interstitial fluid of non-lesional skin reached the LLOQ at approximately 10 h post-dose and maximum concentrations were observed at 18 h post-dose (0.07–1.86 ng/ml, mean C_max_ 0.61 ng/ml; Fig. 3a). In lesional skin, the CP-17 levels steadily increased on day 1, but most subjects did not reach the LLOQ during the whole 24 h sampling period (range 0.09–0.42 ng/ml, mean C_max_ 0.19 ng/ml; Fig. 3b).

On day 14, CP-17 baseline concentrations in dermal interstitial fluid of non-lesional skin were not quantifiable. CP-17 levels showed a steep increase resulting in a peak at around 10 h (0.43–4.1 ng/ml, mean C_max_ 1.00 ng/ml; Fig. 3a). In lesional skin, the CP-17 levels were already quantifiable at baseline (pre-dose), were then transiently dropping to levels below LLOQ after dosing before moderately increasing to a peak at 18 h (0.13–1.53 ng/ml, mean C_max_ 0.68 ng/ml; Fig. 3b).

The cumulated quantities (AUC 0–24 h, Table I) after one day of treatment were 0.854 ± 0.422 ng and 2.142 ± 1.993 ng in lesional and non-lesional skin, respectively (*p* = 0.033). This difference was not statistically significant considering the Bonferroni-adjusted significance level of *p* = 0.0125. Corresponding values after 14 days of treatment were increased (2.768 ± 2.010 ng and 4.439 ± 4.602 ng) but more similar to each other compared to day 1 (*p* = 0.349). In lesional skin, the AUC-increase compared to day 1 was pronounced but did not reach statistical significance (*p* = 0.020) considering Bonferroni. The increase for non-lesional skin was less pronounced (*p* = 0.093). Table I summarizes the AUCs. Notably, this conventional comparison of AUCs does not yet take the factor probe depth into account.

**Table I:** AUCs Derived from Unmodified Data incl. ANOVA Post-hoc Test on AUC and Log(AUC)

Group	Time	N	Variable	Median	Mean	*SD*	Day 1 *vs*. Day 14	L *vs*. NL
**L**	**Day 1**	8	AUC	0.732	**0.854**	*0.422*	***p*** = ***0.020***	***p*** = ***0.033***
**L**	**Day 14**	8	AUC	2.071	**2.768**	*2.010*		*p* = *0.349*
**NL**	**Day 1**	8	AUC	1.705	**2.142**	*1.993*	*p* = *0.093*	
**NL**	**Day 14**	8	AUC	2.521	**4.439**	*4.602*		

Conventional data treatment (values < LLOQ set to LLOQ or LLOQ/2) resulted in a significant increase of the mean profiles, in particular for profiles from lesional skin, which diminished the differences (Figure S1). Thus, when values < LLOQ were substituted by LLOQ/2, the difference found between lesional skin and non-lesional skin on day 1 as well as the increase in lesional skin from day 1 to day 15 did not reach the level of statistical significance (*p* = 0.057 and *p* = 0.053, Suppl. Table S2). When substituted by LLOQ, the level of significance was clearly missed (*p* = 0.111 and *p* = 0.113, Suppl. Table S3).

### Evaluation of Influential Parameters

**Fig. 4:**
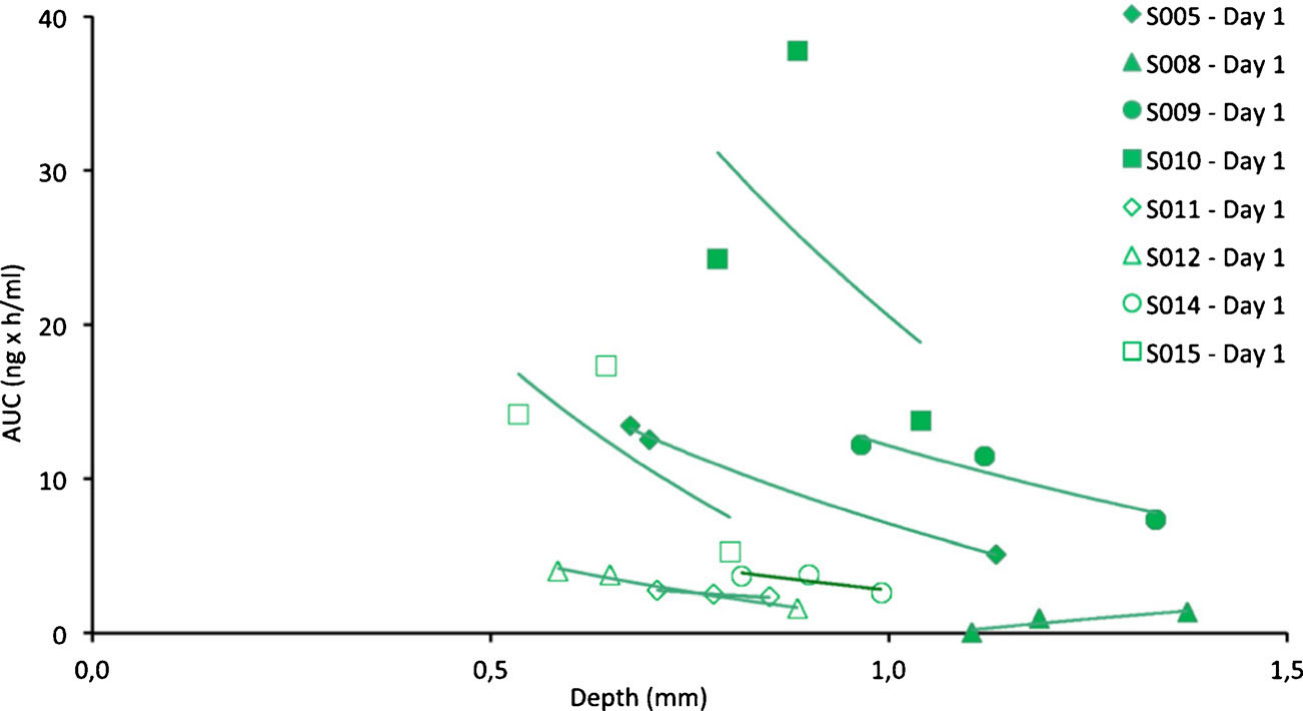
AUC data of non-lesional skin on day 1 plotted *versus* depth. Regression lines are fitted to the AUCs of the 3 adjacent probes in non-lesional skin for each subject. The illustration indicates a relationship between the AUCs and probe depth in 7 of 8 subjects (negative slopes) and also indicates that most of the variability is due to inter-subject variability of CP-17 penetration.

The dependency of AUCs from depth was indicated by a plot of AUCs *versus* probe-depth (Fig. 4). The AUCs of adjacent probe triplets are clearly related by depth (i.e., negative slopes only), while the remaining AUC variability is attributed to the individuals as such (i.e., the inter-subject variability of the skin barrier).

Frequency analysis showed that in 23 out of 30 cases (77%), the deeper probe showed a lower AUC than the more superficial probe. The frequency increased to 89% when the depth-difference was larger than the ultrasound measurement error of 0.2 mm. The paired *t*-test showed a statistically significant difference in AUC (*p* < 0.001) between deeper (2.017 ± 2.689 ng*h/ml) and more superficial probes (2.938 ± 3.024 ng*h/ml).

Finally, the linear mixed effects model identified (i) skin type (lesional *versus* non-lesional, *p* = 0.0158), (ii) time (day 1 *versus* day 14, *p* < 0.0001) and (iii) probe depth (*p* < 0.0001) as statistically significant predictor variables for AUC. The following equations summarize the result of the modelling process:

Non-lesional skin at Day 1:

1 $$ \mathrm{A}\mathrm{U}\mathrm{C}= \exp \left(1.725\hbox{-} 1.737*\ \mathrm{Depth}\right) $$

Non-lesional skin at Day 14:

2 $$ \mathrm{A}\mathrm{U}\mathrm{C}= \exp \left(2.554\hbox{-} 1.737*\ \mathrm{Depth}\right) $$

Lesional skin at Day 1:

3 $$ \mathrm{A}\mathrm{U}\mathrm{C}= \exp \left(1.370\hbox{-} 1.737*\ \mathrm{Depth}\right) $$

Lesional skin at Day 14:

4 $$ \mathrm{A}\mathrm{U}\mathrm{C}= \exp \left(2.199\hbox{-} 1.737*\ \mathrm{Depth}\right) $$

**Fig. 5:**
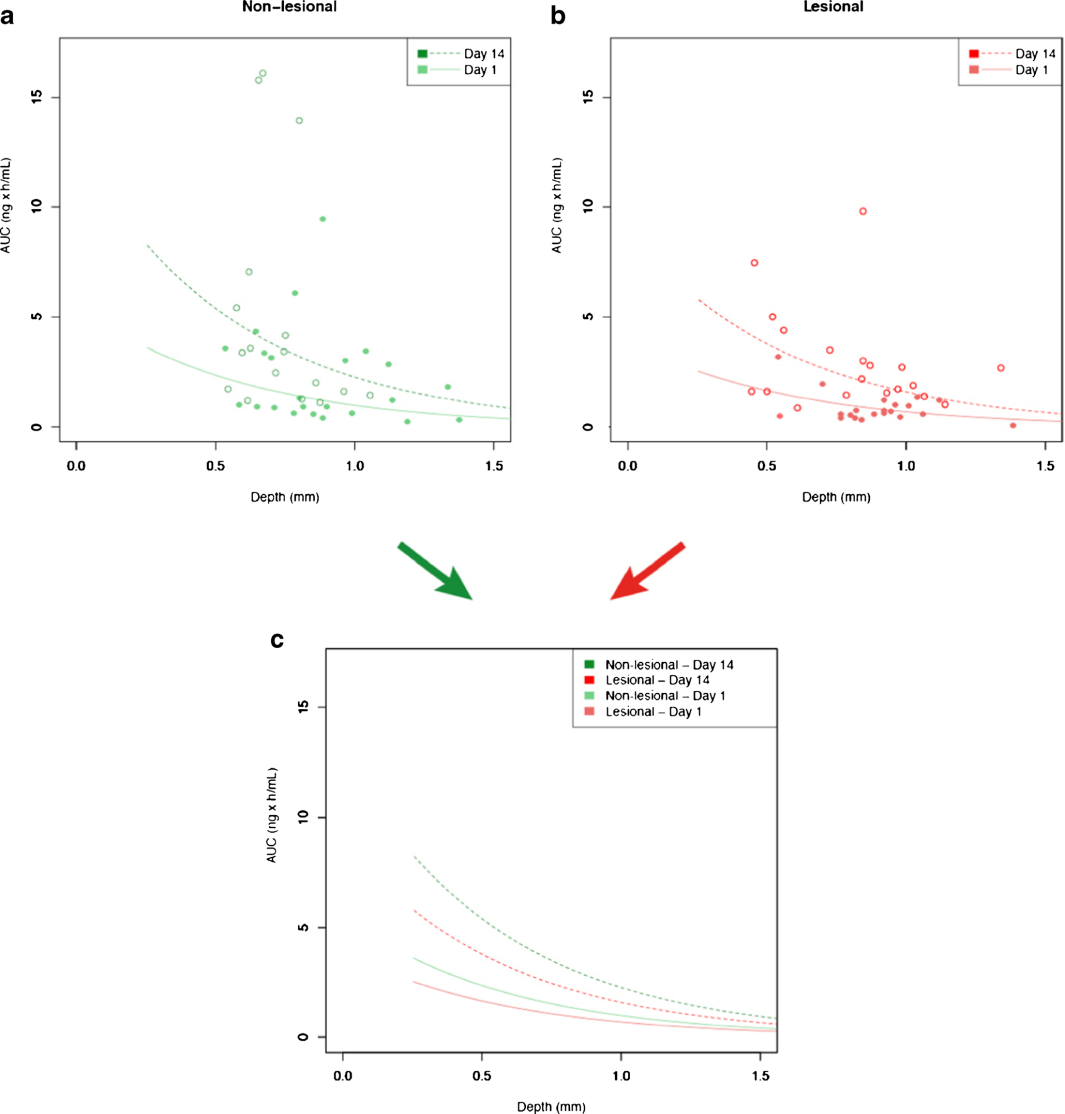
AUC0-24 h for CP-17 in dOFM samples on the days 1 and 14 as a function of depth. (**a**) AUCs of non-lesional skin and (**b**) AUCs of lesional skin; (**c**) All four AUC regression lines from both non-lesional and lesional skin on both day 1 and day 14 (see Eqs. 1–4).

Figure 5 provides a plot of these equations and their fit to the underlying AUC data. This representation shows the differences in AUC between lesional and non-lesional skin both after the 1st dose and the 14th dose, and the AUC increase by daily topical dosing due to the faster absorption.

## Discussion

The intradermal CP-17 concentration profiles assessed in this study revealed a significantly higher penetration of CP-17 into non-lesional skin than into lesional psoriatic skin. We also found that penetration into lesional skin increased with skin healing and that CP-17 did not significantly accumulate in the skin regardless of the skin condition. Thus, by using dOFM, we were able to identify kinetic differences rather than measuring the difference in total quantities only, as has been done in previous studies using classical sampling methods (4, 6). Additionally, we identified dOFM probe depth as a relevant variable to be considered in future topical pharmacokinetic studies with dOFM or dermal microdialysis.

dOFM proved to be suitable for sampling of highly lipophilic drugs because the large openings in the dOFM probe allowed sampling of the total drug concentration without any adsorptive losses. This study provided a CP-17 profile when released from a topically applied low strength cream (0.05% CP-17). In-depth analysis of the AUCs revealed that rather minor differences of 0.2 mm in dOFM probe depths already influence the AUCs observed for CP-17. The identification of probe depth as a relevant variable indicated that the overall methodological variation of dOFM is low. Although the influence of probe depth on kinetics has repeatedly been considered in dermal MD studies (15, 18), no significant effect has been published yet. Methodological differences or the different distribution of predominantly water soluble compounds which have been investigated in these dermal MD studies could have prevented the identification of any impact of probe depth variations. One methodological study found that different depths of the MD probes yielded different AUCs (19) but the depths differences were rather large (1 mm) and thus not representative for standardized use of probes in clinical studies. In contrast, our study assessed the impact of small inevitable depth differences occurring during probe insertion in standardized topical PK studies. We found a statistically significant impact of these small depth differences on the measured CP-17-profiles and this study provided a concentration *versus* depth profile for this glucocorticoid. A similar depth-dependent profile has been shown in the past for topical hydrocortisone by using radio-labelling and slicing methodology (20).

These results indicate that future dOFM/MD studies which are comparing the PK of topical formulations, like topical bioequivalence studies, should consider that (i) probe depths should be precisely measured (by e.g., full length scanning of each probe using a wide angle ultrasound device), (ii) a potential impact of probe depth should be evaluated and (iii) any impact should be adequately taken into account in the representation of the PK outcome variables.

We investigated the kinetic differences of drug concentrations in lesional and non-lesional skin. These kinetic results shed light on the skin penetration properties of a high-potent topical glucocorticoid and on the role of the SC in psoriasis for the absorption of topically applied lipophilic drugs. The penetration of CP-17 into the dermis after the 1st dosing on day 1 was slow as expected and reached C_max_ at 18 h in non-lesional skin. Interestingly, the penetration into lesional skin was even slower and the measured dermal CP-17 concentrations were significantly reduced. This is in agreement with a previous dOFM study that investigated the penetration behavior of the moderately lipophilic topical drug candidate BCT194 (12). One reason for the lower drug levels measured by dOFM in the dermis could be the distance between the dOFM probe and the SC barrier in the inflamed and thus highly blood-perfused dermis, but lower drug levels in lesional skin were also found by tape stripping and skin punch biopsy from *in vitro* and clinical studies (Rony *et al*., unpublished data presented at Groupe de Métabolisme et Pharmacocinétique -Meeting 2011, Paris). Rony *et al*. showed that 70–80% of the penetrated dose has been recovered in the SC in healthy skin, while in psoriatic skin at least 95% of the penetrated dose has been recovered in the SC. Less than 1% of the applied dose reached the deeper skin layers, such as epidermis and dermis, which is the site of drug action. The authors concluded that in psoriasis the hyperkeratosed SC seems to absorb higher amounts of the topical drug and therefore acts as a trap compartment. However, this study assessed drug penetration and distribution only at one single point in time and thus does not allow the investigation of transdermal penetration kinetics to reveal the role of SC in topical drug release. Nevertheless, their data and the results from our study are in contrast to the common assumption that diseased skin is more permeable for topical drugs. Recent reviews of *in vitro* (21) and *in vivo* studies (22) concluded that permeation through damaged skin is modestly increased at least for hydrophilic compounds, but studies on psoriatic skin are scarce. One cited study found that psoriatic and unaffected skin showed similar permeability to hydrocortisone, a glucocorticoid less lipophilic than CP-17 (23). Based on this study it has been suggested that psoriasis might not have a penetration barrier defect or that the penetration of lipophilic compounds is not influenced by damaged skin (22). In conclusion, our dOFM data clearly showed that the permeability of the SC barrier is not impaired for topical drug penetration in psoriasis but may instead act as a trap compartment and thus impact the penetration rate for many of the predominantly lipophilic topical drugs.

The low baseline levels on day 14 of our study indicate that, irrespective of the skin condition, CP-17 does not significantly accumulate in the dermis following repeated dosing. Following the 14th dose, the dermal CP-17 concentrations increased significantly faster in lesional skin, but also somewhat faster in non-lesional skin compared to day 1. For lesional skin, the significantly increased absorption on day 14 can be explained by the ongoing healing of the skin leading to a partial normalization (thinning) of the thickened psoriatic SC. Faster penetration into non-lesional skin on day 14 can be explained by the well-known side effect of prolonged topical glucocorticoid therapy that leads to steroid atrophy of the skin (24–26). Noteworthy, the intradermal CP-17 levels on day 14 did not return to baseline after 24 h due to the fact that the cream on this study day has not been removed after approximately 4 h but remained on the skin.

The clinical effects of a glucocorticoid therapy were confirmed by the significantly decreased total sum scores (TSS) for the treated sites, which were also well demarked as white zones within the plaques. This observation is consistent with the well-known skin blanching response of topical corticosteroids (27).

The intradermal sampling of CP-17 was challenging as indicated by the low concentrations where C_max_ did not exceed the 3-fold LLOQ. In the explorative data analysis we either accepted values below LLOQ or substituted them by a standard value (LLOQ or LLOQ/2) and the results demonstrated that standardized raw data treatment should be used with caution in particular for slow penetrating drugs, as it may bias the data.

A major challenge was the cleaning of the probe insertion sites on day 14 to prevent the contamination of the probes by residual concentrations in the SC. The increased baseline and initial post-dose values in lesional skin on day 14 indicated that the cleaning measures were not sufficient. Therefore, in studies including insertion of probes in topically treated skin, the skin cleaning procedure should be further improved, or Band-Aids should be used during the treatment phase to prevent a carry-over of the drug to skin outside the treatment site when the volunteers are at home. We found considerable inter-subject variability regarding the penetration and the dermal drug levels especially in psoriatic lesions. High inter-subject variability in skin permeability has also been observed clinically (13).

Our study has provided intradermal kinetic data of a topical drug *in vivo* in humans with a time and depth related resolution, when released from a low-strength product. This allows experimental verification of mathematical physiological models for topical drug penetration (28, 29). To support these models, further topical drugs with different lipophilicity and protein binding properties should be investigated. The dOFM approach is well tolerated by healthy subjects and psoriatic patients and it recovers the total drug concentration directly at the site of action. This approach may have a general utility across the entire range of topical dermatological drug products, including early head-to-head testing of formulations in humans when dosed in low quantities as well as head-to-head topical bioequivalence studies of generic *versus* reference listed drug products. Moreover, since dOFM samples represent diluted but otherwise unfiltered dermal interstitial fluid, dOFM samples also include intradermal biomarkers which can be analyzed based on novel highly sensitive low-volume cytokine assays. Hence, future studies can be performed as combined pharmacokinetic and pharmacodynamic studies.

## Conclusion

In conclusion, the dermal pharmacokinetics of highly lipophilic CP-17 released from a commercial low-strength cream in patients *in vivo* was successfully assessed by dOFM and time as well as depth (i.e., spatially) resolved kinetic data were delivered. These data showed that CP-17 does not significantly accumulate in the dermis following repeated topical dosing and revealed a reduced penetration rate of the high-potent glucocorticoid into hyperkeratosed psoriatic skin. Therefore, our data support the assumption that the thickened SC of psoriatic skin acts as trap compartment for lipophilic topical drugs. In terms of methodological advancements our study shows the utility of dOFM to support the clinical development of new drugs by facilitating studies with short treatment duration and low drug amount at early stages with limited preclinical prerequisites. Demonstrating the high spatial resolution of the dOFM sampling probes, our study provides essential information for the proper design and interpretation of topical bioequivalence studies.

## Electronic Supplementary Material

Below is the link to the electronic supplementary material.

Fig. S1 (DOCX 263 kb)

Table S2 (DOCX 262 kb)

Table S3 (DOCX 262 kb)

## Abbreviations

AUC: Area Under the CP-17 concentration time Curve
CP-17: Clobetasol-17-propionate
dOFM: Dermal open flow microperfusion
LLOQ: Lower Limit of Quantification
MD: Microdialysis
SC: Stratum Corneum
TSS: Total Sum Score
