# Supplementary material for: Kinetics of Clobetasol-17-Propionate in Psoriatic Lesional and Non-Lesional Skin Assessed by Dermal Open Flow Microperfusion with Time and Space Resolution
Source: Pharm Res. 2016 Jun 6;33:2229–38. doi: 10.1007/s11095-016-1960-y (PMC4967091; doi:10.1007/s11095-016-1960-y)
Supplement: Supplementary file 1 — (DOCX 263 kb) [file 11095_2016_1960_MOESM1_ESM.docx]

Supplementary Data


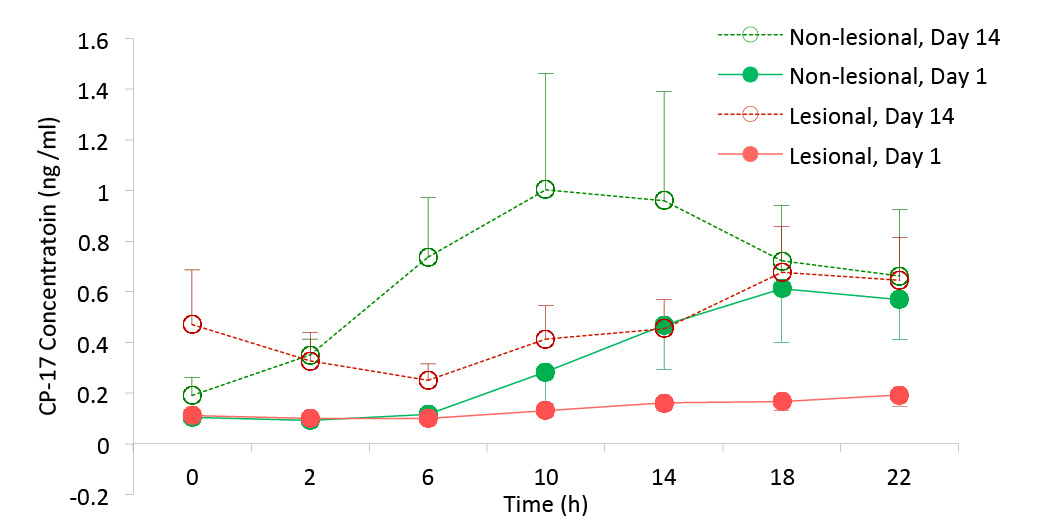


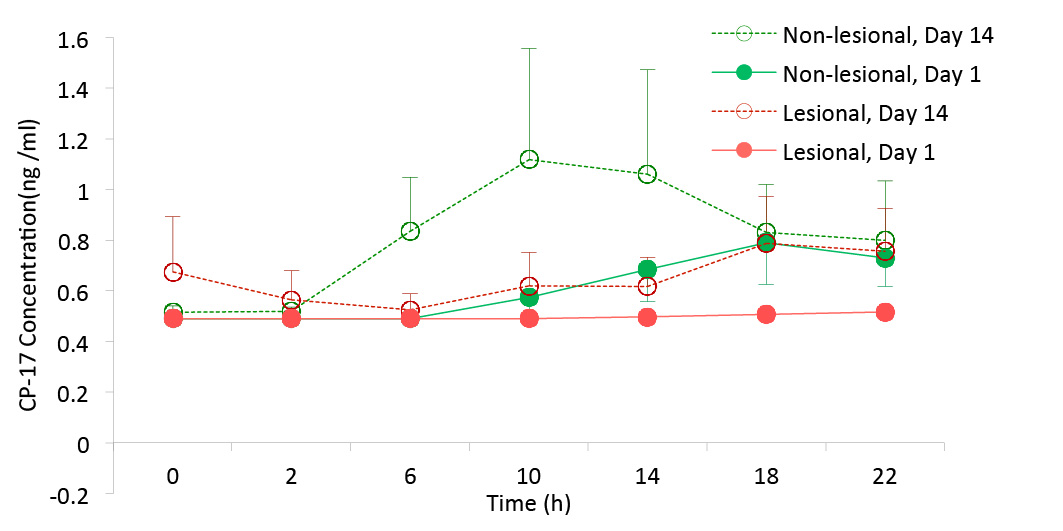


**Figure S1**: Mean CP-17 concentration profiles for lesional and non-lesional skin on Day 1 and Day 14. A) Unmodified data. B) Values <LLOQ replaced by the LLOQ. Data are mean ± sem.
