# Supplementary material for: Kinetics of Clobetasol-17-Propionate in Psoriatic Lesional and Non-Lesional Skin Assessed by Dermal Open Flow Microperfusion with Time and Space Resolution
Source: Pharm Res. 2016 Jun 6;33:2229–38. doi: 10.1007/s11095-016-1960-y (PMC4967091; doi:10.1007/s11095-016-1960-y)
Supplement: Supplementary file 2 — (DOCX 262 kb) [file 11095_2016_1960_MOESM2_ESM.docx]

**Table S2**: AUCs derived from data modified by setting <LLOQ-values to ½ LLOQ incl. ANOVA post-hoc test on AUC and log(AUC)

| **Group** | **Time** | **N** | **Variable** | **Median** | **Mean** | ***SD*** | **Day 1 vs. Day 14** | **L vs. NL** |
| --- | --- | --- | --- | --- | --- | --- | --- | --- |
| **L** | **Day 1** | 8 | AUC | 1.47 | **1.47** | *0.00* | *p=0.053* | *p=0.057* |
| **L** | **Day 14** | 8 | AUC | 1.90 | **2.81** | *1.89* |  | *p=0.605* |
| **NL** | **Day 1** | 8 | AUC | 2.02 | **2.59** | *1.68* | *p=0.544* |  |
| **NL** | **Day 14** | 8 | AUC | 2.15 | **4.14** | *4.74* |  |  |
