# Supplementary material for: Kinetics of Clobetasol-17-Propionate in Psoriatic Lesional and Non-Lesional Skin Assessed by Dermal Open Flow Microperfusion with Time and Space Resolution
Source: Pharm Res. 2016 Jun 6;33:2229–38. doi: 10.1007/s11095-016-1960-y (PMC4967091; doi:10.1007/s11095-016-1960-y)
Supplement: Supplementary file 3 — (DOCX 262 kb) [file 11095_2016_1960_MOESM3_ESM.docx]

Table S3: AUCs derived from data modified by setting <LLOQ-values to LLOQ incl. ANOVA post-hoc test on AUC and log(AUC)

| **Group** | **Time** | **N** | **Variable** | **Median** | **Mean** | ***SD*** | **Day 1 vs. Day 14** | **L vs. NL** |
| --- | --- | --- | --- | --- | --- | --- | --- | --- |
| **L** | **Day 1** | 8 | AUC | 2.94 | **2.94** | *0.00* | *p=0.113* | *p=0.111* |
| **L** | **Day 14** | 8 | AUC | 3.00 | **3.79** | *1.39* |  | *p=0.597* |
| **NL** | **Day 1** | 8 | AUC | 3.24 | **3.70** | *1.33* | *p=0.570* |  |
| **NL** | **Day 14** | 8 | AUC | 3.13 | **5.00** | *4.30* |  |  |
